# Can Interactions between Timing of Vaccine-Altered Influenza Pandemic Waves and Seasonality in Influenza Complications Lead to More Severe Outcomes?

**DOI:** 10.1371/journal.pone.0023580

**Published:** 2011-08-23

**Authors:** Utkarsh J. Dang, Chris T. Bauch

**Affiliations:** 1 Department of Mathematics and Statistics, University of Guelph, Guelph, Ontario, Canada; 2 Department of Ecology and Evolutionary Biology, Princeton University, Princeton, New Jersey, United States of America; Yale University, United States of America

## Abstract

Vaccination can delay the peak of a pandemic influenza wave by reducing the number of individuals initially susceptible to influenza infection. Emerging evidence indicates that susceptibility to severe secondary bacterial infections following a primary influenza infection may vary seasonally, with peak susceptibility occurring in winter. Taken together, these two observations suggest that vaccinating to prevent a fall pandemic wave might delay it long enough to inadvertently increase influenza infections in winter, when primary influenza infection is more likely to cause severe outcomes. This could potentially cause a net increase in severe outcomes. Most pandemic models implicitly assume that the probability of severe outcomes does not vary seasonally and hence cannot capture this effect. Here we show that the probability of intensive care unit (ICU) admission per influenza infection in the 2009 H1N1 pandemic followed a seasonal pattern. We combine this with an influenza transmission model to investigate conditions under which a vaccination program could inadvertently shift influenza susceptibility to months where the risk of ICU admission due to influenza is higher. We find that vaccination in advance of a fall pandemic wave can actually increase the number of ICU admissions in situations where antigenic drift is sufficiently rapid or where importation of a cross-reactive strain is possible. Moreover, this effect is stronger for vaccination programs that prevent more primary influenza infections. Sensitivity analysis indicates several mechanisms that may cause this effect. We also find that the predicted number of ICU admissions changes dramatically depending on whether the probability of ICU admission varies seasonally, or whether it is held constant. These results suggest that pandemic planning should explore the potential interactions between seasonally varying susceptibility to severe influenza outcomes and the timing of vaccine-altered pandemic influenza waves.

## Introduction

Both seasonal and pandemic influenza are associated with a considerable burden of disease, in the form of absenteeism, hospitalizations, intensive care unit (ICU) admissions, and deaths [1]. Severe complications can occur even in patients without chronic health conditions [2]. Complications are often the result of secondary bacterial infections, and the delay between primary influenza and the diagnosis of subsequent bacterial infections means that the primary influenza infection is not always confirmed [3]. Data from the influenza pandemics of 1918, 1957 and 1968 are consistent with secondary bacterial pneumonia causing the majority of influenza-associated deaths [4]. Pandemic influenza can be associated with a higher burden of disease than seasonal influenza, if only because more individuals become infected during a pandemic due to lower levels of natural immunity in the population, as compared to typical seasonal influenza [5].

As with previous influenza pandemics, the 2009 H1N1 pandemic imposed a significant disease burden [6]. Pulmonary complications were common, with primary influenzal pneumonia and acute respiratory distress syndrome in adults and secondary bacterial pneumonia in children [7]. Secondary pneumococcal infections were often a factor in severe and fatal cases of influenza [8]. However, unlike in previous pandemics, immunization programs may have played a mitigating role, despite late introduction of the vaccine. The use of a vaccine against pandemic influenza for the first time ever suggests that immunization will form a part of mitigation plans for future influenza pandemics. However, as always, it remains necessary to address how best to design and execute large-scale immunization programs in the face of uncertainties.

Seasonal influenza is characterized by strong seasonal variation in incidence, generally surging in the winter months in temperate regions [1]. Severe outcomes such as influenza-related hospitalizations and deaths also peak in winter [9], [10]. In comparison, although influenza pandemics may be influenced by seasonality, they do not always follow the same pattern: spring, fall and winter waves have all occurred in past pandemics [11]. One possible explanation for this difference is that the widespread host susceptibility that accompanies an antigenically novel strain means an outbreak can occur even when seasonal factors do not support its transmission.

The cause of seasonality in seasonal influenza is debated [12]. Suggested contributors include seasonal variation in host health, school attendance, ambient temperature, indoor/outdoor absolute humidity, and ultraviolet (UV) radiation intensity [12]–[15]. Some of these observations have led to a hypothesis that innate host susceptibility varies seasonally, enabling seasonal outbreaks to occur. Impairments of the antimicrobial peptide (AMPs) systems that respond to influenza infection are caused by very low levels of 25-hydroxy-vitamin D [25(OH)D] in the winter months [16]. Vitamin D levels vary with seasonal trends in UV radiation levels and are therefore highest in August and lowest in February (since most Vitamin D is obtained through sun exposure, not diet). This implies heightened host susceptibility in late fall, winter, and early spring [17].

Susceptibility to secondary bacterial infections from influenza also varies seasonally, and exposure to UV-B radiation is known to reduce the risk of invasive pneumococcal disease (pneumonia, bacteremia, and meningitis) [18]. Seasonality in susceptibility to secondary bacterial infections suggests that influenza outcomes might be more severe when peak influenza incidence aligns with peak susceptibility to secondary bacterial infections. Immunization alters the susceptibility of the host population to influenza infection and may thereby affect the timing of pandemic influenza peaks [19]. Therefore, a pandemic immunization program may mitigate a fall wave and reduce the total number of influenza infections. However, at the same time, it may push some influenza incidence into winter months where an influenza infection is more likely to cause severe secondary bacterial infections. Because of these two factors have competing effects on the number of severe outcomes, it is not clear *a priori* what their net impact will be. Thus, it is worthwhile to investigate the potential for such a result.

Mathematical models can be used to address such questions because they offer an opportunity to explore a wide range of scenarios, including not only scenarios close to what actually happened but also scenarios that might have happened, or that may happen in the future. A significant amount of modeling has addressed the topic of mitigation strategies for influenza pandemics [20]–[30]. Some of this work investigates the potential role of interventions or co-infections in creating multiple pandemic waves [23]–[25], [29]. Many models only track a single outcome measure–the incidence of influenza infection–and hence cannot predict serious outcomes like hospitalizations and ICU admissions. However, severe outcomes from influenza are very important for decision-making given that most individuals recover from infection without experiencing severe outcomes. Several models do include such outcomes, but they tend to assume the probability of severe outcomes per influenza infection does not vary seasonally [20], [27] and hence they cannot be used to explore interactions between vaccine-altered timing of pandemic influenza waves and seasonal variation in susceptibility to severe outcomes such as caused by secondary bacterial infections.

Here, we use a mathematical model to investigate conditions under which a pandemic immunization program could increase the number of ICU admissions by increasing influenza infections in months where infection is more likely to lead to ICU admission. The model assumes seasonality in both influenza transmission rates and susceptibility to influenza infection, as well as seasonality in host susceptibility to ICU admission caused by influenza. The probability that an influenza infection leads to an ICU admission, as a function of time of year, is estimated using data from the 2009 H1N1 influenza pandemic. We also compare the predicted number of ICU admissions when the probability of ICU admission per incident influenza infection varies seasonally to when it does not. The latter simplifying assumption is implicit to most pandemic models, hence this comparison allows us to determine its impact on model predictions.

## Methods

Our SIRS (Susceptible-Infectious-Recovered-Susceptible) compartmental model of influenza transmission divides the population into Susceptible, Infectious and Recovered/Immune categories (see Text S1). Despite its simplifying assumptions, the SIRS model and its variants have been shown to capture features of real influenza outbreaks, such as observed epidemic curves and seasonal patterns [23], [25], [31], [32]. Susceptible individuals become infectious at a rate equaling an infection rate parameter 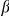 times the number of susceptible individuals 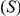 times the proportion of the population that is infectious 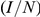. The infection rate 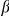 varies seasonally to reflect seasonal variation in both influenza transmissibility and susceptibility to influenza infection [33], [34]. The amplitude of seasonality is controlled by 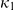 (amplitude of seasonality in transmission due to changes in school attendance over the school year) and 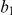 (amplitude due to any other sources of seasonality in transmission and susceptibility to infection). Individuals flow from infectious (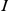) to immune (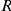) at a rate 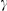 (where 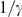 is the mean infectious period). The average value of 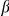 over a year, 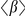, can be determined from the basic reproduction number 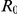 according to 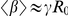 (see Text S1). Individuals are immunized at a rate 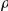 and the vaccine has efficacy 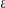. Efficaciously vaccinated individuals are moved from the 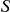 to the 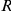 compartment. Individuals are born into the susceptible compartment at rate 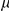 per capita, and individuals in any compartment die from all causes at rate 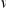 per capita. The strain is introduced at time 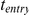 during the summer, and a proportion R(0) of individuals are assumed initially immune due to previous vaccination and infection (e.g. Spring wave).

As in many previous models, we model antigenic drift as a flow of individuals from immune (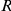) to susceptible (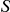) due to waning immunity at a rate 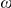 (where 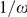 is the average duration of immunity). This gradual loss of immunity represents the effects of antigenic drift within a population. However, new cross-reactive antigenic variants can also be introduced to a population by case importation, meaning a large proportion of the population must be immediately re-classified as susceptible. This may happen within a pandemic year: for instance, sequential variants of the virus have been posited as one possible reason for the repeated waves such as observed in the 1918 pandemic [25], [35]. Hence at the end of December (during the holiday travel period), a proportion 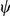 of recovered individuals were transferred to the susceptible compartment to represent importation of a novel cross-reactive strain into the population. (We also explored 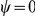 in sensitivity analysis.) We note that modelling the effects of importing a cross-reactive strain would not be captured adequately through the 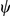 parameter if co-circulation of the two strains were occurring; however, in our simulations, the fall wave caused by a first strain is over by the time the cross-reactive strain is introduced and hence this is not an issue. Parameter values and their sources appear in Table S1 [36]–[45]. The model was simulated in Matlab [46].

The number of new ICU admissions per week (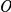) was taken as 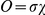, where 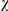 is number of incident influenza cases per week (derived from the SIRS model) and 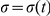 is the probability that an influenza infection in week 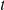 causes ICU admission. The function 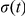 varied seasonally according to a modified sinusoidal function (Table S2). This function was fitted to data on lab-confirmed influenza cases and ICU admissions attributable to influenza infection from the 2009 H1N1 pandemic [47]–[49].

To assess the impact of parameter uncertainty and understand how model dynamics vary across a range of plausible parameter values, we conducted a probabilistic (Monte Carlo) sensitivity analysis. To ensure model realism, the sensitivity analysis also excluded parameter combinations that gave rise to unrealistic dynamics,. We defined lower and upper plausible ranges for the parameters 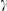, 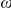, 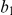, 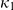, 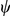, 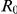, 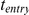 and the initial condition 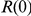 (Table S1). We conducted many realizations, and for each realization we drew samples from these ranges for each parameter, simulated the model equations, and generated outcomes for each sampled set of parameter values. However, to ensure realism and consistency with experience from past pandemics, we excluded any realizations (1) that yielded a summer peak in the following calendar year, (2) that did not end up producing normal seasonal influenza peaks (i.e. a single peak in fall, winter or spring) in any of the three following calendar years, or (3) that produced closely spaced peaks in both December and February of the pandemic year (these simulations were excluded because we wished to simplify the analysis by restricting attention to simulations where there was only one peak per season). These three criteria were applied in the absence of an immunization program. Fall was defined as September to November, winter as December to February, and spring as March to May.

Approximately 1,000 simulations survived this filtering Monte Carlo algorithm and were used to generate results for the prevalence and incidence of influenza infection as well as the incidence of ICU admissions, in the absence of an immunization program. From this, we computed the total number of ICU admissions from the fall to the following summer. To evaluate the impact of vaccination, we ran the same set of simulations in the presence of an immunization program. We analyzed a total of 30 immunization program designs based on a subset of the following combinations of characteristics: (1) Starting September 1, October 1, or November 1, (2) lasting 1, 2, 3, 4, 5 or 6 months, (3) vaccinating 10%, 20%, 40% or 60% of the population (Table S3). However we only report results for seven profiles denoted ‘A’ through ‘G’ vaccinating 40% of the population (Table 1) since the other 23 profiles either produced qualitatively similar results or produced trivial results because most vaccine ended up being administered after the fall wave.

**Table 1:** Description of different vaccination profiles.*

Vaccination Profile:	A	B	C	D	E	F	G
Month of initiation	Sep	Sep	Sep	Sep	Oct	Oct	Oct
Length of vaccination program (months)	3	2	1	3	3	2	1
Distribution per month	1/3, 1/3, 1/3	1/2, 1/2	1	1/2, 1/4, 1/4	1/3, 1/3, 1/3	1/2, 1/2	1

*A 40% vaccination rate was assumed for results presented in main text. The distribution per month indicates what portion of the 40% were vaccinated each month. So, in vaccination profile D, in September, half of the targeted population gets immunized with a fourth of the targeted population getting immunized in each of the following 2 months.

## Results

**Figure 1:**
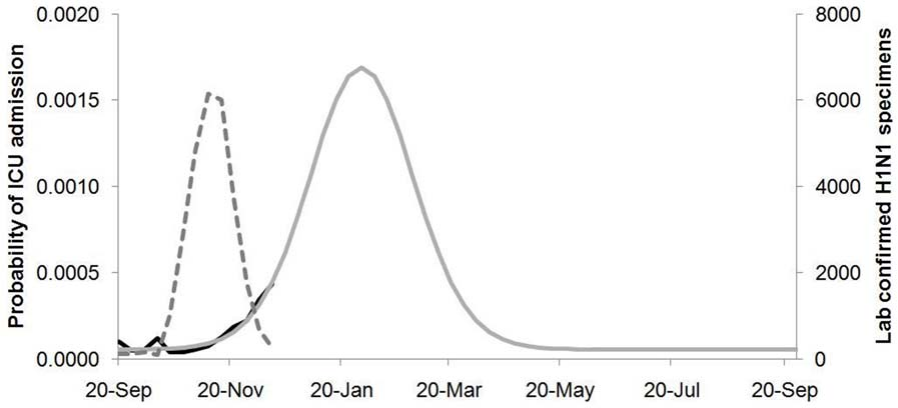
Probability of ICU admission per influenza infection, estimated from the H1N1 pandemic of 2009. Data from the H1N1 pandemic of 2009 (black line) was fit to our seasonally varying function (Table S2) and extrapolated (solid grey line). Weekly H1N1 positive specimens (dashed grey line) is on the secondary vertical axis.

The best fit of the seasonally-varying probability of ICU admission per influenza infection to the data appears in Figure 1. This figure shows a clear increase in the probability of ICU admission per new influenza infection as winter progresses, even as the total number of influenza infections declines as the fall wave ends. The probability of being admitted to ICU due to an influenza infection in the extrapolated model is highest in the months of January and February, which is consistent with what we would expect based on the literature describing peak susceptibility to secondary bacterial infections in these months. Only data to mid-December were used to fit the model because influenza-attributable ICU admissions and lab-confirmed influenza cases became too few after that point to support fitting. Also, numbers reported after late December had new counts based on retrospective analysis of the spring and fall waves rather than being based on new case reports alone, which could have confounded the results.

**Figure 2:**
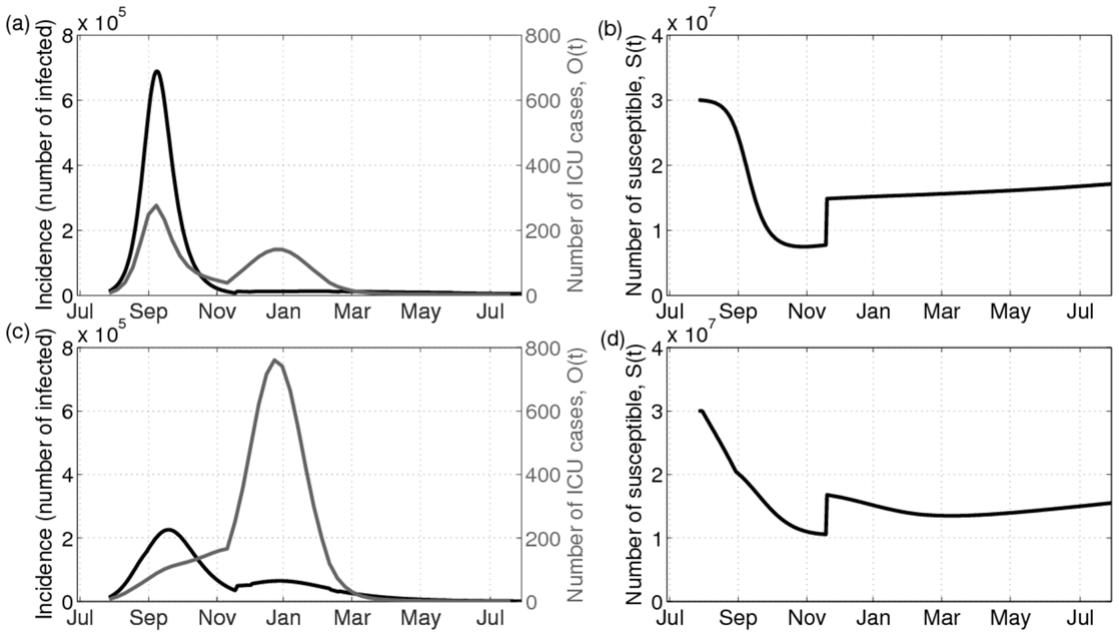
Example where vaccination increases the number of ICU admissions. A typical run where the number of ICU admissions increased due to vaccination for Profile C for 40% vaccination rate. (a), infected incidence (black) and number of ICU admissions (grey) over time without vaccination (top panel) and (c), with vaccination (bottom panel). (b), time series of the number of susceptible individuals without (top panel) and (d), with vaccination (bottom panel). Vaccination leads to an increase in the number of ICU admissions by increasing the number of susceptibles available for another wave leading to higher incidence and higher morbidity. Parameter values: 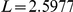 years, 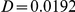 years, 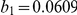, 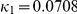, 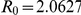, 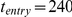, 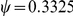, 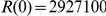 and 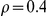.

Figure 2 shows how influenza incidence, number of ICU admissions, and susceptibility to influenza infection evolve over time for a typical realization of the simulation, with and without a vaccination program. Without a vaccination program, there is a single peak in infection prevalence in October, which also corresponds to a peak in the number of ICU admissions (Figure 2a). The pool of susceptible individuals is rapidly depleted by the first wave, but in December it begins increasing again due to the effects of case importation, and thereafter climbs gradually due to recruitment of new susceptible individuals through births and antigenic drift (Figure 2b). This increase in susceptibility is not sufficient to cause a second wave, although it does cause a second peak in the number of ICU admissions (Figure 2a). The addition of a vaccination program (profile C from Table 1) in this example alters these dynamics considerably. Immunization significantly reduces the number of individuals infected by the first pandemic wave, which still peaks in October, but at a considerably lower level (Figure 2c versus Figure 2a). This reduces the number of ICU admissions in October (Figure 2c). However, due to the mitigating effects of the vaccination program, the pool of susceptible individuals is larger by the start of the following January (Figure 2d versus Figure 2b). As a result, there is a second pandemic wave in January when the probability of severe outcomes is highest, which causes a large peak in ICU admissions (Figure 2c). The net result is that the total number of ICU admissions from Fall to Summer is higher with the vaccination program than without it. In this simulation, the combination of higher susceptibility due to importation of a cross-reactive strain December and implementation of an immunization program in early Fall are enough to increase total susceptibility in January to the point where a second wave is possible.

**Figure 3:**
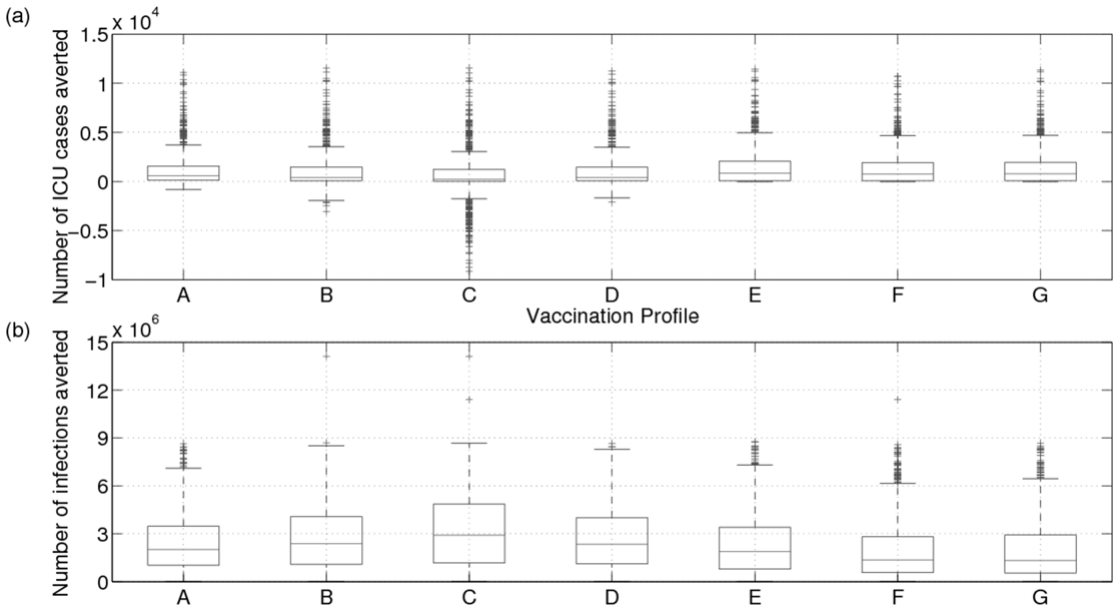
ICU admissions averted (top) and infections averted (bottom) for different vaccination profiles. Boxplots of ICU admissions and infections averted with different vaccination profiles for 40% vaccination rate. Vaccinating a larger proportion of the population in advance can lead leads to a lower number of infections in total but a higher number of ICU admissions. Points are drawn as outliers 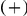 if they are larger than 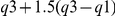 or smaller than 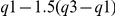, where q1 and q3 are the 25th and 75th percentiles, respectively.

Under certain vaccination programs, an increase in net ICU admissions can occur. For example, we found that vaccination profile C leads to an increase in ICU admissions in 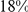 of the approximately 1,000 realizations used for the analysis (Figure 3). This is more than any other vaccination profile, and–surprisingly–despite the fact that profile C also averts more influenza infections than any other profile. Profile C, which covers 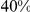 of the population in September, vaccinates more individuals prior to the Fall wave than do Profiles A, B or D, in which vaccination also starts in September but is spread over two or three months and continues during the Fall wave. Similarly, profiles A, B and D increase ICU admissions in 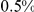, 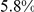 and 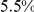 of realizations, respectively. In comparison, profiles E–F which start immunizing in October always avert influenza infections without causing a net increase in ICU admissions (Figure 3). This occurs because in most realizations, the fall wave is already under way when vaccination starts in October. Hence, profiles E–F prevent fewer influenza infections and consequently shift fewer susceptible individuals to the winter months when the risk of severe outcomes from influenza infection is higher.

In our baseline parameters, the value of 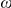 (within-population antigenic drift) was too small to cause an increase in susceptibility that, when combined with the mitigating effects of a vaccine, would increase ICU admissions. However, if 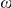 is increased sufficiently (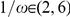 months), similar effects are observed as for large values of 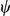, and an immunization program can increase the total number of ICU admissions even if 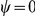 (Figure S1). Hence, a vaccination program can cause adverse outcomes whether susceptibility increases suddenly due importation of a cross-reactive strain, or gradually due to within-population antigenic drift. In the special case 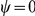 (no importation of cross-reactive strain) and 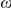 is at its smaller baseline value, no realizations yielded an increase in the number of ICU admissions for any of the vaccination profiles (Figure S2). In the case 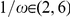 months (rapid antigenic drift), and 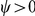, we again found that some vaccination programs (especially profile C) could lead to more ICU admissions (Figure S3). Hence, our model predicts an increase in ICU admissions due to a vaccination if importation of a cross-reactive strain occurs or if antigenic drift within the population is sufficiently fast.

**Figure 4:**
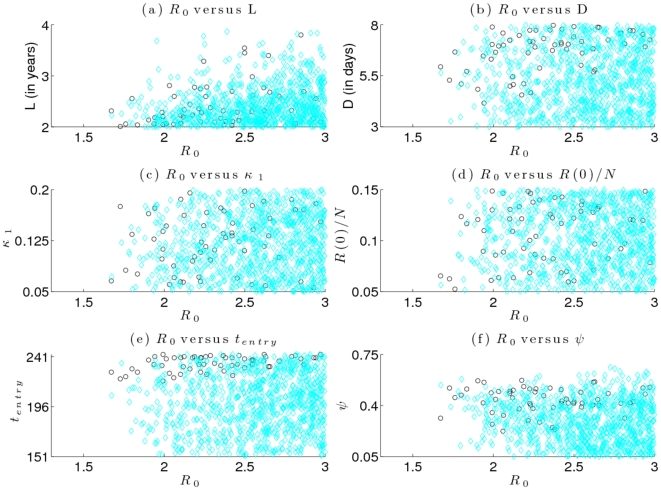
Scatter plots of realizations where ICU admissions increased or decreased due to vaccination, as a function of various parameters. Parameters where vaccination caused a higher (black circles) or lower (blue diamonds) number of ICU cases for Profile B for 40% vaccination rate. There is some evidence for clustering near the upper constraint for the entry time parameter. 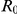 (horizontal axis in all panels) values smaller than approximately 1.7 did not give rise to simulations that passed our filtering criterion, hence the lack of data for these values of 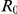. Longer duration of infectiousness (smaller 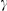), more rapid antigenic drift (larger 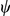), or when the entry time is later in the summer (larger 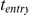) seem more conducive to resulting in an increase in the number of ICU admissions.

To understand which parameter combinations are most likely to increase the number of ICU admissions, we compared scatter plots of realizations where vaccination led to more ICU admissions to scatter plots of realizations where it led to fewer ICU admissions, using vaccination profile B as an example (Figure 4). These scatter plots suggest that vaccination is more likely to cause an increase in ICU admissions when the duration of infectiousness is longer (smaller 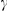), antigenic drift is more rapid (larger 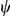), or when the entry time is later in the summer (larger 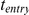). For a given 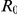, a longer duration of infectiousness will spread out the epidemic curve, increasing incidence in the winter months and hence ICU admissions. A later entry time or more antigenic drift before January likewise mean that susceptibility, and hence incidence, is higher in the winter months. Thus, in general, when processes that tend to shift susceptibility to winter months are already present, implementation of a vaccination program is more likely to cause a further increase in susceptibility in winter and hence a net increase in ICU admissions. As the duration of the immunization program decreases from 3 months (Profile A) to 1 month (Profile C), the range of values for 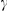, 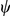 and 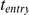 for which vaccination caused more ICU admissions become widened (results for Profiles A and C not shown).

For profiles A–D, vaccination increases the number of ICU admissions every time there is a transition from a fall wave without vaccination, to fall and winter waves with vaccination (Table 2). In this case, the vaccination program creates a small second wave at a time where the risk of ICU admission is higher. This also happens very frequently when vaccination causes a transition from a fall-and-spring wave to a fall-and-winter wave, or from a fall wave to a fall-and-spring wave (Table 2). In this case, vaccination adds susceptibility before the start of the winter and thus leads to an earlier second wave (in winter instead of spring), which thereby results in more ICU admissions. Hence, ICU admissions are most likely to increase if vaccination increases susceptibility enough to cause a second wave in the winter months. These are often the outliers in Figure 3.

**Table 2:** Number of realizations where vaccination led to a higher number of ICU cases because of a transition of pandemic waves.*

Vaccination Profile	A	B	C	D
Fall-and-spring to fall-and-winter	0 (0)	0 (0)	18 (18)	0 (0)
Fall to fall-and-winter	0 (0)	1 (1)	6 (6)	1 (1)
Fall to fall-and-spring	0 (0)	4 (5)	14 (14)	2 (4)
Fall to fall-and-summer	0 (0)	2 (3)	12 (22)	1 (1)

*The values in parenthesis are the total number of results observed out of 1000 included simulations for the specific vaccination profile for 40% vaccination rate.

It is instructive to compare the predicted number of severe outcomes in a model with seasonally varying susceptibility to severe outcomes (“seasonal estimator”) to a model where susceptibility to severe outcomes is the same in all months (“average estimator”). If the predictions are significantly different, this provides a rationale for pandemic influenza models to include interactions between seasonally varying susceptibility to severe outcomes and timing of vaccine-altered influenza pandemic waves. We made this comparison by replacing the seasonally varying probability of ICU admission per influenza infection with a constant probability, parameterized using the same data from the 2009 H1N1 pandemic (see Text S1). Across all vaccine program profiles, the model predictions vary significantly depending on whether the average or seasonal estimator is used (Table 3). The average estimator predicts somewhat more ICU admissions than the seasonal estimator, in the absence of a vaccination program. This occurs because the probability of ICU admission per influenza infection in the fall months (when a pandemic peak occurs) according to the seasonal estimator is lower than the probability used in the average estimator, which was obtained by averaging the frequency of ICU admissions over the period of the whole pandemic including later months when ICU admission was a more likely outcome of infection. However, the seasonal estimator predicts more ICU admissions averted by vaccination programs (Table 3). Also, the variation in ICU admissions averted by vaccination is much higher across the different vaccination profiles for the seasonal estimator than for the average estimator. For example, more ICU admissions are averted under the seasonal estimator than the average estimator for profile A, but the difference is quite low for profile C. Results are similar for 10%, 20% and vaccine 60% coverage (results not shown). For the case of a pathogen that is more virulent than the 2009 H1N1 pandemic strain was, leading to a uniformly higher probability of ICU admission per week, we note that the predictions remain the same: the seasonal estimator predicts a higher number of ICU admissions averted by vaccination programs (results not shown).

**Table 3:** Comparison between ICU estimates (standard deviations) from the average and seasonal estimators.*

Vaccination Profile ( 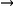 )	A	B	C	D	E	F	G
Number of ICU admissions on average using the average function without vaccination	4391 (756)	4391 (755)	4392 (756)	4392 (755)	4388 (755)	4389 (754)	4392 (756)
Number of ICU admissions on average using the seasonal function without vaccination	3443 (2882)	3439 (2871)	3438 (2864)	3443 (2882)	3431 (2877)	3436 (2876)	3447 (2883)
Number of ICU admissions averted on average using the average function	341 (255)	378 (278)	448 (321)	373 (265)	329 (280)	270 (259)	277 (268)
Number of ICU admissions averted on average using the seasonal function	1192 (1662)	1081 (1740)	538 (2309)	1084 (1703)	1454 (1863)	1334 (1700)	1359 (1752)

*For simulations of approximately 1000 runs each for the two strain case for 40% vaccination rate. The mean of the number of ICU admissions on average using both the average and seasonal function vary a little bit across vaccination profiles due to runs (from particular combinations of parameters) resulting in a flag that made our pandemic filtering criteria exclude it.

We carried out a similar comparison of average and seasonal estimators for the case where 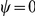, corresponding to no importation of a cross-reactive strain in December (Table S4). We found that, as for the 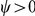 case, both the predicted number of ICU admissions with vaccination, and the number of ICU admissions averted by a vaccination program, differed widely depending on whether the average or seasonal estimator is used. Hence, even when vaccination cannot cause an increase in ICU admissions by increasing incidence in winter months, the predicted number of ICU admissions averted by vaccination programs can vary considerably depending on whether or not seasonality in the probability of severe outcomes is taken into account.

## Discussion

Here we used a model to explore potential interactions between seasonally varying susceptibility to severe outcomes from influenza infection and the timing of influenza pandemic waves as modified by vaccination programs. We found that vaccination programs could increase ICU admissions by increasing susceptibility to infection in winter months if (1) there is a pre-existing tendency toward higher susceptibility in early winter due to antigenic drift or importation of a cross-reactive strain, and (2) vaccination occurs in advance of the fall pandemic wave. We found that scenarios where vaccination began during the pandemic wave instead of before it did not increase ICU admissions, simply because these programs do not protect as many individuals by virtue of starting later, and hence they do not increase susceptibility enough in the winter. An increase in ICU admissions occurred only when we allowed for introduction of a cross-reactive variant in December or when antigenic drift was sufficiently fast. However, even when we did not allow this to happen, the predicted number of ICU admissions averted by vaccination varied significantly depending on whether we allowed the probability of ICU admission per influenza infection to vary seasonally or whether we force it to be constant, suggested the current approach of assuming the same probability of severe outcomes across all months may be too limited.

These results suggest that fall pandemic waves may be a blessing in disguise in some respects, since under a fall wave, the majority of individuals are infected and attain natural immunity before the time of year when the risk of severe outcomes from influenza infection is highest. However, the number of severe outcomes due to a fall pandemic wave are not necessarily small in absolute terms. For example, the Fall wave of the 1918 pandemic was the deadliest on historical record, and the cause of this may have been secondary bacterial infections [4]. Baseline host susceptibility to severe secondary bacterial infections has likely evolved over the past century.

Here we focused on ICU admissions as a marker of severe outcome measures. Although risk of ICU admission is likely to correlate with risk of death and risk of hospitalization, an expanded model including a broader array of outcomes may yield different predictions. Our discussion about the causes of higher severity in winter months focused on secondary bacterial infections, but there are other reasons why health outcomes of influenza may be worse in the winter months such as greater crowding of ICUs and demand for resources such as extra-corporeal membrane oxygenation (ECMO) due to seasonal peaks in non-influenzal pneumonia and other seasonal viral infections.

We used a simplified method of capturing the effects of importing a cross-reactive strain by changing the number of susceptible individuals in December, rather than explicitly modelling two strains. The results of a two-strain model can differ significantly, especially if both strains are co-circulating or differ in their antiviral resistance. However, in our case this will have little effect since the first pandemic wave was finished by early December in most realizations and hence co-circulation would not occur. The degree of natural cross-protection is reflected in how many individuals are moved to the susceptible compartment. Therefore, the impact of this simplified approach is minimal.

Previous work on the impact of pandemic interventions on the susceptibility profile of a population over time has identified conditions where early application of an intervention (in particular, antiviral drugs) can lead to a second wave by boosting susceptibility in later months [23]. This previous work did not include severe outcomes as a model output. However, their findings combined with ours do suggest that widespread prophylactic use of antiviral drugs in fall or early winter might have a similar effect of increasing the total number of ICU admissions. In fact, we expect the effect to be greater because antiviral drugs only reduce susceptibility while they are being taken, whereas vaccines confer longer term protection.

Here, we relied on data from the 2009 H1N1 pandemic. The 2009 pandemic did not have a 2010 winter or spring wave in northern countries so we extrapolated the fall data to obtain the probability of ICU admission in winter and spring. However, we expect many of our results to generalize for any pandemic in which the probability of severe outcomes per influenza infection varies seasonally. It is also important to note that there is potential for confounding in the model estimates for case severity since other factors in additional to seasonal variation in severity may influence the probability of severe complications. For instance, large subpopulations may have co-morbidities that predispose them to severe outcomes, and these subpopulations may experience the highest incidence of influenza infection at a time of year that is different from that of the rest of the population [50]. Despite this, our model fits showed that there is not much change in the probability of a severe outcome in the summer when the disruptive effects of this kind of confounding variable would be strongest, which agrees well with what was observed in the first wave of the pandemic of 2009 [51].

Including other features of influenza transmission might change our quantitative predictions. For instance, explicitly modelling co-circulation of a second drug-resistant strain, modelling the circulation of relevant bacterial infections, or adding age structure, social structure, or other heterogeneities could influence model predictions. The predictions of a model with feedback between bacterial transmission and influenza transmission (where each influences the other) in particular might diverge considerably from our model predictions. However, most of these potential extensions should not change the fact that vaccination can delay the peak of an epidemic and thus increase influenza incidence in winter months, and that this might increase the number of ICU admissions under certain conditions. We also expect the finding that seasonal estimators and average estimators give rise to very different predicted ICU admissions will be robust under potential model extensions.

Effective pandemic planning requires accounting for various possible scenarios that may unfold in a real pandemic. Use of a vaccine in future pandemics is a likely scenario, given its prominent role in the 2009 H1N1 pandemic. Future increases in manufacturing efficiency could also mean pandemic vaccines are available well in advance of future fall pandemic waves. Our research has shown that the predicted impact of vaccine programs on the incidence of severe outcomes due to influenza infection can be different–sometimes even opposite–to the impact on total influenza infections, if susceptibility to severe outcomes is modelled as a seasonally varying function. More research is required to understand how including interactions between seasonal susceptibility to severe outcomes and vaccine-altered pandemic waves in models can change which vaccine programs are predicted to be optimal from the public health perspective. Future models should include the kinds of severe outcomes that are often at the front of policy-makers' minds, such as physician office visits, hospitalizations and ICU admissions, and they should also explore whether their conclusions change if the probability of severe outcomes is allowed to vary seasonally.

## Supporting Information

Text S1 **Detailed information on model equations and parameterization.**
(PDF)

Figure S1
**Boxplots of ICU admissions averted and infections averted for different vaccination profiles when** 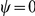 **and** 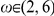 **months (40% vaccination rate).** Points are drawn as outliers 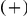 if they are larger than 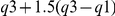 or smaller than 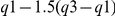, where q1 and q3 are the 25th and 75th percentiles, respectively.(PDF)

Figure S2
**Boxplots of ICU admissions averted and infections averted for different vaccination profiles when** 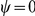 **(40% vaccination rate).** Points are drawn as outliers 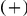 if they are larger than 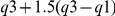 or smaller than 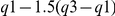, where q1 and q3 are the 25th and 75th percentiles, respectively.(PDF)

Figure S3
**Boxplots of ICU admissions averted and infections averted for different vaccination profiles when** 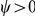 **and** 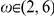 **months (40% vaccination rate).** Points are drawn as outliers 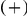 if they are larger than 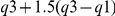 or smaller than 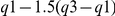, where q1 and q3 are the 25th and 75th percentiles, respectively.(PDF)

Table S1 **Parameter values.**
(PDF)

Table S2 **Estimates of seasonal variation in probability of ICU admission per influenza infection with 95% confidence intervals.**
(PDF)

Table S3 **List of immunization programs (vaccination profiles).**
(PDF)

Table S4
**Comparison between ICU estimates (standard deviations) from the average and seasonal functions **(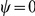)**.**
(PDF)
