# Supplementary material for: Can Interactions between Timing of Vaccine-Altered Influenza Pandemic Waves and Seasonality in Influenza Complications Lead to More Severe Outcomes?
Source: PLoS One. 2011 Aug 23;6(8):e23580. doi: 10.1371/journal.pone.0023580 (PMC3160314; doi:10.1371/journal.pone.0023580)
Supplement: Text S1 — Detailed information on model equations and parameterization. (PDF) [file pone.0023580.s001.pdf]

Supporting Text

The SIR model equations are:

$$\frac{dS}{dt} = -\rho S + \psi R - \frac{\beta SI}{N} + \omega R + \mu N - \nu S \tag{1}$$

$$\frac{dI}{dt} = \frac{\beta SI}{N} - \gamma I - \nu I \tag{2}$$

$$\frac{dR}{dt} = \rho S - \psi R + \gamma I - \omega R - \nu R \tag{3}$$

where S, I and R are the number of susceptible, infected and removed individuals respectively. Here, N is the population size, β is the transmission rate, ω is the rate of loss of immunity, γ is the recovery rate, μ and ν are the crude birth rate and crude death rate respectively, ρ is the vaccination rate and was nonzero only over the months where a vaccination program was being applied. For those months, it was set according to the vaccine coverage and duration. For instance, for a profile covering 40% of the population over 2 months, ρ was set to 20% (0.2) per month or equivalently, 2.4/year. ψ is the percentage of people from the removed compartment transferred to the susceptible section due to circulation of a second strain of the pandemic virus on December 20th. Furthermore, the initial number of infected was chosen to be 50000 to reflect a previous wave of infection in the spring.

School term forcing and the effect of holidays is recognized to be important in modeling influenza [33, 34] and so we incorporate that in our model. The infection rate function β is a product of a school term function κ that incorporates seasonality in transmission caused by changes in school attendance over the school term, and a sinusoidal function θ that implicitly incorporates both other sources of seasonality in susceptibility to influenza infection, and seasonality in the transmissibility of the influenza virus:

$$\kappa = \kappa_0 (1 + \frac{\tau}{365} \kappa_1) \tag{4}$$

when school is in session and

$$\kappa = \kappa_0 (1 - \frac{365 - \tau}{365} \kappa_1) \tag{5}$$

when school is out. Here, $\tau = 82$ is the total number of holidays except for weekends during school terms, κ_0 is the baseline rate and κ_1 is the amplitude of the seasonality in transmission. The sinusoidal function

θ is:

$$\theta = b_0(1 + b_1 cos(2\pi t)) \quad (6)$$

where b_0 and b_1 are the baseline rate and amplitude of seasonality in susceptibility to influenza infection respectively, and t is time in years. The number of new severe outcomes per week was estimated by $O = \sigma\chi$ where χ is the weekly incidence of infecteds computed from the compartmental model and σ is the weekly probability of a severe outcome as a result of pandemic influenza:

$$\sigma = d_2 + e^{d_0(1+d_1 cos(\frac{2\pi t}{365}))} \quad (7)$$

where d_0, d_1 and d_2 were estimated by fitting the above function to epidemiological data from the H1N1 pandemic of 2009 [49].

Assuming the weekly number of positive H1N1 specimens (recorded by provincial laboratories) as a proxy for the prevalence of H1N1 in the Canadian population at any given time, we used its product with an appropriate scaling factor such that the total prevalence of H1N1 in the population was 30% [47, 48]. We calculated the probability of a case being admitted to ICU conditional on this weekly measure of H1N1 cases in the population. We hypothesized that the time dependent relationship of the number of ICU admissions as a result of influenza would follow a seasonal relationship based on some recent vitamin D, UV radiation and immune system literature as discussed in the introduction. The data was fit to a sinusoidal ($d_0(1+d_1 cos(\frac{2\pi t}{365}))$) and exponential ($d_2+e^{d_0(1+d_1 cos(\frac{2\pi t}{365}))}$) function both with and without an intercept respectively using the curve fitting tool cftool from Matlab [46] and subsequently extrapolated to get a probabilistic relationship for the entire year. The sinusoidal function did not capture the trend of the data as well as the exponential function. Adjusted R^2, root mean squared error and the Akaike Information Criterion were used to choose the better model (Table S2, Figure 1) and the exponential function with the intercept gave a better fit according to these values.

Since we used data from the H1N1 pandemic of 2009, these fitted values correspond to the 2009 H1N1 situation only, and for a different pandemic parameter space, would have to be refit. The average estimator probability was estimated by averaging out the weekly probability calculated from the epidemiological data from the H1N1 pandemic of 2009 [49]. The differential equations (1-3) were solved numerically using ode23, an implementation of an explicit Runge-Kutta (2, 3) pair of Bogacki and Shampine in Matlab [46].

We also define

$$R_0 \approx \frac{\langle\beta\rangle}{\gamma} \tag{8}$$

where the brackets denote the time average over one year. Therefore, since $\beta(t) = \kappa(t)\theta(t)$,

$$R_0 = \frac{\langle\kappa(t)\rangle\langle\theta(t)\rangle}{\gamma} \tag{9}$$

But since,

$$\langle\kappa(t)\rangle = \kappa_0 \tag{10}$$

and

$$\langle\theta(t)\rangle = b_0 \tag{11}$$

we obtain

$$R_0 = \frac{\kappa_0 b_0}{\gamma} \tag{12}$$

We set $\kappa_0 = 1$ without loss of generality, and for each sampled value of R_0 and γ we obtain b_0 from

$$b_0 = \frac{R_0\gamma}{\kappa_0} = R_0\gamma \tag{13}$$
