# Supplementary material for: Can Interactions between Timing of Vaccine-Altered Influenza Pandemic Waves and Seasonality in Influenza Complications Lead to More Severe Outcomes?
Source: PLoS One. 2011 Aug 23;6(8):e23580. doi: 10.1371/journal.pone.0023580 (PMC3160314; doi:10.1371/journal.pone.0023580)
Supplement: Figure S2 — Boxplots of ICU admissions averted and infections averted for different vaccination profiles when (40% vaccination rate). Points are drawn as outliers if they are larger than or smaller than , where q1 and q3 are the 25th and 75th percentiles, respectively. (PDF) [file pone.0023580.s003.pdf]

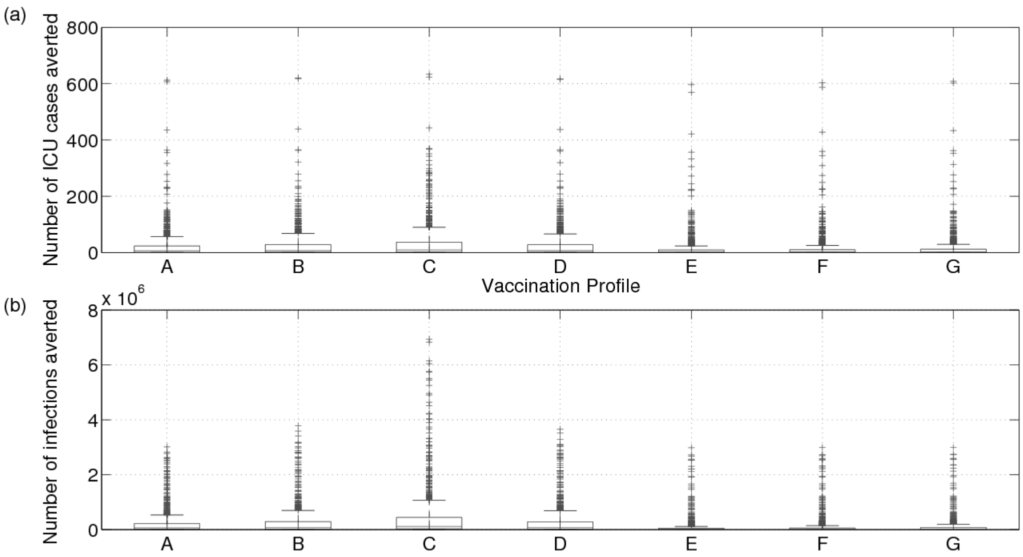

Figure S2. Boxplots of ICU admissions averted and infections averted for different vaccination profiles when $\psi = 0$ (40% vaccination rate). Points are drawn as outliers (+) if they are larger than $q3 + 1.5(q3 - q1)$ or smaller than $q1 - 1.5(q3 - q1)$, where q1 and q3 are the 25th and 75th percentiles, respectively.
