# Supplementary material for: Can Interactions between Timing of Vaccine-Altered Influenza Pandemic Waves and Seasonality in Influenza Complications Lead to More Severe Outcomes?
Source: PLoS One. 2011 Aug 23;6(8):e23580. doi: 10.1371/journal.pone.0023580 (PMC3160314; doi:10.1371/journal.pone.0023580)
Supplement: Table S1 — Parameter values. (PDF) [file pone.0023580.s005.pdf]

Table S1. Parameter values*.

Parameter	Definition	Range/Values	Source
$1/\omega$	Average duration of immunity	$(2, 4)$ years	[36]
$1/\gamma$	Average infectious period	$(3, 8)$ days	[36, 42, 44]
μ	Crude birth rate of the population per 1000 people	10.6	[45]
ν	Crude death rate of the population per 1000 people	10.6	Assumed similar to μ for convenience
ρ	Vaccination rate	40%	Assumption
ψ	Percentage of people from the removed section transferred to the susceptible section due to circulation of a second strain of the influenza virus at the start of the Christmas break	$(5, 75)$	Assumption
t_{entry}	Day of introduction of strain during summer	$(152, 243)$	Randomly sampled from days from June to the end of August
κ_0	Baseline contribution of school term effects to transmission	1	Assumption without loss of generality
κ_1	Amplitude of seasonality in transmission due to school term	(0.05, 0.2)	Assumption
$b_0 = \gamma R_0/\kappa_0$	Baseline contribution of non-school factors to transmission and susceptibility		Derived
b_1	Amplitude of seasonality in transmission and susceptibility due to other sources	(0.01, 0.10)	Assumption
R_0	Basic reproduction number	(1.3, 3)	[37–41, 43]
ϵ	Efficacy of vaccine	0.75	Assumption

*Parenthetical values indicate input ranges for Monte Carlo filtering algorithm.
