# Supplementary material for: Can Interactions between Timing of Vaccine-Altered Influenza Pandemic Waves and Seasonality in Influenza Complications Lead to More Severe Outcomes?
Source: PLoS One. 2011 Aug 23;6(8):e23580. doi: 10.1371/journal.pone.0023580 (PMC3160314; doi:10.1371/journal.pone.0023580)
Supplement: Table S2 — Estimates of seasonal variation in probability of ICU admission per influenza infection with 95% confidence intervals. (PDF) [file pone.0023580.s006.pdf]

Table S2. Estimates of seasonal variation in probability of ICU admission per influenza infection with 95% confidence intervals

Equation	Adjusted R^2	AIC Criterion	Root Mean Squared Error	Fitted Values
$e^{(d_0(1+d_1 cos\frac{2\pi t}{365}))}$	0.84	-218.93	4.97×10^{-5}	$d_0 = -9.4(-9.87,-8.93)$; $d_1 = 0.28(0.20,0.36)$
$d_2 + e^{(d_0(1+d_1 cos\frac{2\pi t}{365}))}$	0.94	-230.02	3.00×10^{-5}	$d_0 = -10.65(-11.36,-9.95)$; $d_1 = 0.3978(0.32,0.48)$; $d_2 = 5.14\times10^{-5}$ (fixed at bound)
