# Supplementary material for: Can Interactions between Timing of Vaccine-Altered Influenza Pandemic Waves and Seasonality in Influenza Complications Lead to More Severe Outcomes?
Source: PLoS One. 2011 Aug 23;6(8):e23580. doi: 10.1371/journal.pone.0023580 (PMC3160314; doi:10.1371/journal.pone.0023580)
Supplement: Table S3 — List of immunization programs (vaccination profiles). (PDF) [file pone.0023580.s007.pdf]

Table S3. List of immunization programs (vaccination profiles)*

Month of initiation	Length of program	Proportion of ρ per month
September, October, November	$1, 2, 3, 4, 5, 6$ months	equally distributed each month
September, October, November	3	$1/2, 1/4, 1/4$
September, October, November	4	$1/3, 1/3, 1/6, 1/6$
September, October, November	4	$1/2, 1/6, 1/6, 1/6$
September, October, November	6	$1/4, 1/4, 1/8, 1/8, 1/8, 1/8$

*Vaccinating more individuals in advance of pandemic wave leads to a higher probability of increasing the number of ICU admissions due to vaccination. These profiles were run at 10, 20, 40 and 60% rates respectively.
