# Supplementary material for: Can Interactions between Timing of Vaccine-Altered Influenza Pandemic Waves and Seasonality in Influenza Complications Lead to More Severe Outcomes?
Source: PLoS One. 2011 Aug 23;6(8):e23580. doi: 10.1371/journal.pone.0023580 (PMC3160314; doi:10.1371/journal.pone.0023580)
Supplement: Table S4 — Comparison between ICU estimates (standard deviations) from the average and seasonal functions (). (PDF) [file pone.0023580.s008.pdf]

Table S4. Comparison between ICU estimates (standard deviations) from the average and seasonal functions ($\psi = 0$)*

Vaccination Profile	A	B	C	D	E	F	G
Number of ICU admissions on average using the average function with vaccination	3523 (265)	3523 (265)	3523 (265)	3523 (265)	3523 (265)	3523 (265)	3523 (265)
Number of ICU admissions on average using the seasonal function with vaccination	1310 (99)	1310 (99)	1310 (99)	1310 (99)	1310 (99)	1310 (99)	1310 (99)
Number of ICU admissions averted on average using the average function	31 (60)	40 (75)	61 (123)	39 (74)	13 (44)	14 (44)	16 (45)
Number of ICU admissions averted on average using the seasonal function	22 (48)	25 (51)	33 (62)	25 (51)	14 (44)	15 (45)	16 (46)

*For simulations of around 1000 runs each for the one strain simulation for 40% vaccination rate.
